# Supplementary material for: Comparative multiomics analysis of cell physiological state after culture in a basket bioreactor
Source: Sci Rep. 2022 Nov 23;12:20161. doi: 10.1038/s41598-022-24687-4 (PMC9686226; doi:10.1038/s41598-022-24687-4)
Supplement: Supplementary file 1 — Supplementary Information 1. [file 41598_2022_24687_MOESM1_ESM.zip › raw data/Transcriptomic raw data/6.Enrichment/2.KEGG/TE1vsTC1/ALL/src/csab04060.html]

csab04060


- csab:103241278

- Down regulated genes

103241278(-4.29901042433954)

- csab:103246725

- Up regulated genes

103246725(2.36022198629957)

- csab:103225819

- Down regulated genes

103225819(-1.75376010187244)

- csab:103242551

- Down regulated genes

103242551(-2.00566883576666)

- csab:103236305

- Up regulated genes

103236305(1.72662479914397)

- csab:103237005

- Down regulated genes

103237005(-1.92426444229972)

- csab:103219268

- Down regulated genes

103219268(-3.50421803072857)

- csab:103223920

- Down regulated genes

103223920(-1.66241974207187)

- csab:103235305

- Up regulated genes

103235305(2.47926657358393)

- csab:103226390

- Up regulated genes

103226390(2.68260263702081)

- csab:103242198

- Down regulated genes

103242198(-2.54075217125675)

- csab:103225587

- Down regulated genes

103225587(-1.86427910916187)

- csab:103221402

- Up regulated genes

103221402(1.05314063148687)

- csab:103235798

- Down regulated genes

103235789(-1.64077436097819)

- csab:103235798

- Down regulated genes

103235789(-1.64077436097819)

- csab:103235798

- Down regulated genes

103235789(-1.64077436097819)

- csab:103242710

- Down regulated genes

103242710(-1.6025804891077)

- csab:103242710

- Down regulated genes

103242710(-1.6025804891077)

Close
